# Investigating the Pharmacological Impact of Atosiban, an Oxytocin Receptor Antagonist, on Bladder and Prostate Contractions Within OBESE and Non-Obese Rats

**DOI:** 10.3390/biomedicines13092097

**Published:** 2025-08-28

**Authors:** Masroor Badshah, Jibriil Ibrahim, Nguok Su, Penny Whiley, Sarah M. Turpin-Nolan, Khaled A. Elnahriry, Ralf Middendorff, Michael Whittaker, Betty Exintaris

**Affiliations:** 1 Hudson Institute of Medical Research, Monash University, Clayton, VIC 3168, Australia; 2 Monash Institute of Pharmaceutical Sciences, Parkville, VIC 3052, Australia; 3 Hull York Medical School, University of Hull, Hull HU6 7RX, UK; 4 Department of Pharmaceutical Sciences, College of Pharmacy, Alfaisal University, Riyadh 11533, Saudi Arabia; 5 Institute of Anatomy and Cell Biology, Justus-Liebig-University, 35390 Giessen, Germany

**Keywords:** lower urinary tract symptoms, overactive bladder, benign prostatic hyperplasia, oxytocin receptor antagonist

## Abstract

**Background/Objectives:** Lower urinary tract symptoms (LUTS), such as frequency, urgency, nocturia, and urge incontinence, are commonly linked to overactive bladder (OAB) and benign prostatic hyperplasia (BPH). Oxytocin receptor (OXTR) upregulation has been proposed to enhance bladder and prostate contractility, while obesity is a recognized risk factor for LUTS, OAB, and BPH. This study aimed to investigate whether the OXTR antagonist atosiban attenuates spontaneous and oxytocin-induced contractions in bladder and prostate tissues from obese and non-obese rats. **Methods:** Bladder and prostate tissues were obtained from obese and non-obese rats and studied in in vitro organ bath preparations. The effects of atosiban (1 µM and 10 µM) on spontaneous contractility and oxytocin-induced responses were examined. Immunohistochemistry was performed to evaluate OXTR expression in the bladder. **Results:** Atosiban significantly reduced spontaneous contractions in the bladder (*p* < 0.0001 in obese; *p* < 0.01 in non-obese) and prostate (*p* < 0.01 in obese; *p* < 0.0001 in non-obese). Oxytocin-induced bladder contractions were significantly increased in obese rats but were attenuated by atosiban at 10 µM (*p* < 0.05), an effect absent in non-obese rats. Immunohistochemical analysis confirmed elevated OXTR expression in both epithelial and stromal compartments of the bladder in obese rats (*p* < 0.05). **Conclusions:** These findings indicate that oxytocin contributes to bladder and prostate hypercontractility, particularly in obesity. Targeting OXTR with atosiban may represent a novel therapeutic strategy for the management of LUTS, OAB, and BPH.

## 1. Introduction

Lower urinary tract symptoms (LUTS) is a broad clinical term encompassing urinary issues such as increased frequency, urgency, nocturia, and incontinence. These symptoms are frequently attributed to overactive bladder (OAB) and benign prostatic hyperplasia (BPH), both of which involve changes in smooth muscle tone within the bladder and prostate [1,2,3]. OAB is typically age-related and is defined by involuntary detrusor contraction leading to urgency, often with or without urge incontinence [1]. BPH, on the other hand, involves a non-malignant enlargement of the prostate due to the hyperplasia of epithelial and stromal components, leading to bladder outlet obstruction and storage symptoms [4,5]. Obesity is increasingly recognized as a significant and independent risk factor for LUTS, OAB, and BPH [6,7,8]. Obesity contributes to metabolic syndrome (MetS), a cluster of conditions including insulin resistance, dyslipidemia, hypertension, and visceral obesity. MetS has been associated with the development and progression of LUTS, especially in men over the age of 60 [9,10,11,12]. Despite this, the precise mechanisms by which obesity influences bladder and prostate function remain incompletely understood. Current pharmacological treatments for LUTS/OAB include antimuscarinic agents, β3-adrenoceptor agonists, tricyclic antidepressants, and hormone therapies. BPH is commonly treated with α1-adrenoceptor blockers, 5α-reductase inhibitors, and phosphodiesterase type 5 (PDE-5) inhibitors [13,14,15,16,17,18]. While effective in many patients, these therapies can cause significant side effects, such as dry mouth, constipation, sexual dysfunction, and mood disturbances, limiting long-term compliance [19]. As a result, there is a growing need for novel therapeutic targets with improved efficacy and tolerability. Recent studies have pointed to the oxytocin (OT) signaling pathway as a potential therapeutic target in lower urinary tract function. OT, a nonapeptide hormone produced in the hypothalamus and released by the posterior pituitary, exerts its physiological effects through the oxytocin receptor (OXTR), a G-protein-coupled receptor expressed in various peripheral tissues, including the bladder and prostate [20,21,22]. Upon activation, OXTR triggers the phospholipase C-inositol triphosphate (PLC-IP3) pathway, increasing intracellular calcium and promoting smooth muscle contractions [23]. Evidence from both animal and human studies suggests that OT may modulate myogenic tone in the lower urinary tract, although the extent of its influence and potential pathophysiological role in obesity-related LUTS remains unclear [24,25,26,27,28].

Emerging evidence also points to a complex interplay between the hypothalamic–pituitary–gonadal (HPG) axis and lower urinary tract function. Testosterone, which declines with age and is often suppressed in obesity and metabolic syndrome, plays a critical role in maintaining prostate growth, smooth muscle tone, and overall bladder function [29,30]. Hypogonadism has been associated with worsening LUTS, potentially through changes in autonomic regulation and smooth muscle reactivity [31]. Notably, oxytocin production is also regulated, in part, by hypothalamic inputs and may be influenced by the hormonal environment, suggesting a potential hormonal–neuroendocrine link in LUTS pathophysiology [32]. However, the interaction between testosterone, oxytocin signaling, and lower urinary tract dysfunction, particularly in the context of obesity, remains poorly understood.

Atosiban, a synthetic desamino-oxytocin analogue, was selected for the present study as a pharmacological tool to investigate OXTR function. It acts as a potent competitive antagonist at the OXTR and blocks vasopressin V1a receptors, thereby inhibiting IP_3_-mediated calcium release and downstream smooth muscle contractions [33,34]. Clinically, atosiban is approved for the treatment of preterm labor and has demonstrated superior tolerability compared to β-agonist tocolytics [35,36]. Its well-characterized pharmacokinetics, established safety profile, and dual receptor antagonism provide both translational relevance and an opportunity to explore potential vasopressin-mediated contributions to bladder and prostate contractility. Previous ex vivo studies have shown that atosiban can significantly reduce spontaneous contractile activity in human prostate tissue [37] and abolish minimal OT-induced contractions in human bladder strips [38], supporting its suitability for functional studies in the lower urinary tract.

Therefore, the primary aim of this study was to assess whether pharmacological inhibition of oxytocin receptor using atosiban could modulate spontaneous and oxytocin-induced contractions in bladder and prostate tissues from obese and non-obese rats. This could offer insights into a novel therapeutic approach for managing LUTS, particularly in obesity-associated cases.

## 2. Material and Methods

### 2.1. Animal Ethics

Ethical approval for access to Sprague Dawley male obese rats (*n* = 6) was granted by the Ethics Committee, Animal House, Parkville (Ref. No. 19717), and for non-obese rats (*n* = 6) it was granted by the Ethics Committee, Monash Animal Research Platform, Clayton (Ref. No. 00000). Factors such as age, diet, housing, and strain were standardized across groups. The obese group (*n* = 6) was fed a semi-pure high-fat diet, formulated to be equivalent to Research Diets D12451, for 12 weeks to induce obesity. Although full metabolic profiling was not performed, the pathophysiological relevance of diet-induced obesity remains well supported. Recent studies demonstrated that high-fat feeding alone is sufficient to trigger key metabolic disturbances, particularly through the enhanced intestinal production of C16:0 ceramides, which contribute to systemic insulin resistance and lipid abnormalities. These findings validate the use of dietary models to mimic obesity-associated metabolic dysfunctions. In contrast, the non-obese control group (*n* = 6) was maintained on a standard laboratory diet [33,34]. In addition, not all rats were pre-treated with any medications prior to the study.

### 2.2. Tissue Collection

Rats (10–12 weeks old) were euthanized via CO_2_ inhalation. The bladder and ventral lobe of the prostate (VLP) were dissected, cleaned, and cut into strips of equal mass for organ bath studies.

### 2.3. Reagent Preparation

Stock solutions (10^−2^ M) of oxytocin (OT) and atosiban (Sigma Aldrich, St. Louis, MO, USA) were prepared in distilled water and diluted in Krebs–Henseleit solution (Ph 7.4 mM: NaCl 118.1, KCl_4_ 69, KH_2_PO_4_ 1.2, NaHCO_3_ 25.0, D (+) glucose 11.7, MgSO_4_·7H_2_O 1.1, CaCl_2_ 2.50) for organ bath use.

### 2.4. Organ Bath Experiments

Bladder and prostate tissue strips (*n* = 6/group) were mounted under 0.5–1 g tension and equilibrated for 60 min. Spontaneous contractions were recorded, followed by oxytocin exposure (1 nM to 100 µM in 10 min intervals), with and without atosiban (1 µM and 10 µM) pre-incubation. Experiments were conducted unblinded. Tissues unresponsive to 20 mM KCl were excluded.

### 2.5. Tissue Viability

Tissue viability was assessed both before and after each experiment by administering 20 mM potassium chloride (KCl). The resulting contractile response was recorded, and only tissues exhibiting robust KCl-induced contractions were included in the analysis. This approach is widely used to validate tissue responsiveness and contractile integrity [35].

### 2.6. Rationale for Drug Concentration

The concentrations of oxytocin and atosiban used in this study were chosen based on prior published studies that demonstrated the effective modulation of target receptors at these doses without inducing adverse effects. These concentrations ensure both relevance and safety for the experimental design [36,37,38].

### 2.7. Data Acquisition and Analysis

Contractile activity was recorded using Chart Pro v7.3.8 and exported to Excel for normalization against peak KCl-induced responses. The analyzed parameters included frequency, amplitude, and integral, defined as the area under the curve (AUC) of the tension trace. The integral (AUC) reflects the total contractile effort over time and provides a comprehensive measure of overall smooth muscle activity. Statistical analyses were performed using GraphPad Prism 9 [Graph Pad Prism version 9.0.1 for Windows (GraphPad Software, La Jolla, CA, USA)], employing two-way ANOVA with Tukey’s post hoc test, unpaired *t*-tests, and EC_50_ calculations where applicable. A *p*-value of less than 0.05 was considered statistically significant.

### 2.8. Immunohistochemistry

Bladder (*n* = 6) and prostate (*n* = 6) tissue sections (4 µm thick) were deparaffinized and rehydrated through xylene and graded alcohols. Antigen retrieval was performed using a low pH buffer at 98 °C for 30 min to expose epitopes. To block non-specific binding, a Fab fragment of donkey anti-mouse IgG was applied. Tissues were then incubated with primary antibodies against the oxytocin receptor (rabbit polyclonal) and smooth muscle actin (mouse monoclonal), alongside isotype controls.

Following washing steps, species-specific fluorescent secondary antibodies (Alexa Fluor 488 and 647) were added to visualize the targets. Cell nuclei were counterstained with DAPI. To minimize tissue autofluorescence, slides were treated briefly with Sudan Black B [Sigma, St. Louis, MO, USA, # 199664-25G]. Finally, sections were mounted using a Prolong Gold antifade reagent (Invitrogen, Waltham, MA, USA, Cat # P36934) and cover slipped for fluorescence microscopy.

### 2.9. Image Acquisition and Quantification

After immunostaining, bladder and prostate tissue sections were imaged using a fluorescence microscope under identical exposure conditions to ensure consistency across samples. Fluorophores were visualized as follows: AF488 (oxytocin receptor, Thermo Fisher Scientific, Waltham, MA, USA) at 1000 ms, AF647 (smooth muscle actin, Abcam, Cambridge, UK) at 590 ms, and DAPI (nuclei, Sigma-Aldrich, St. Louis, MO, USA) at 120 ms. Images were analyzed using OlyVIA software (version 2.9.1, Olympus), and quantitative measurements were performed using QuPath (version 0.3.2). Specific regions of interest were manually selected using the ink tool to ensure consistency across samples, and mean fluorescence intensity values (±SD) were recorded. Data were organized using Microsoft Excel and graphically represented using GraphPad Prism (version 9.0.1). Normality was assessed using the Shapiro–Wilk test, and comparisons between groups were conducted using a two-tailed unpaired *t*-test. Statistical significance was defined as *p* < 0.05, and all values were expressed as mean ± standard deviation (SD).

## 3. Results

### 3.1. Organ Bath Findings

To investigate the effect of oxytocin (OT) and its antagonist atosiban on bladder and prostate contractility, organ bath experiments were conducted using tissues from age- and sex-matched obese and non-obese rats (*n* = 6 per group).

#### 3.1.1. Tissue and Body Weights

The mean ± SEM weights of whole bladders were 0.1 ± 0.01 g for both obese and non-obese rats. Prostate weights were 1.5 ± 0.2 g in obese rats and 1.3 ± 0.1 g in non-obese rats. Whole-body weights were significantly higher in obese rats (580.6 ± 23.1 g) compared to non-obese rats (401.8 ± 15.4 g).

#### 3.1.2. Tissue Viability Confirmed via Potassium Chloride

To confirm tissue viability, all bladder and prostate strips were exposed to 20 mM KCl at the beginning and end of each experiment. Tissues that failed to contract were excluded from analysis. In all included samples, KCl elicited strong and consistent contractions, indicating preserved smooth muscle functionality. No observable differences in contractile amplitude or area under the curve (AUC) were noted between obese and non-obese tissues. While these data are not shown graphically, the consistency of responses across preparations confirmed the reliability of the experimental model.

#### 3.1.3. Effects of Atosiban on Spontaneous Contractions

**Figure 1:**
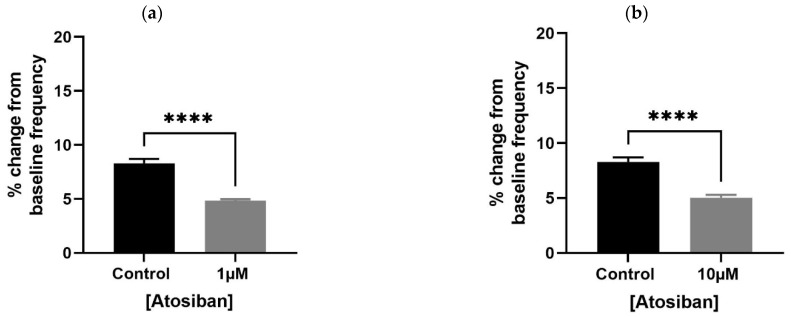

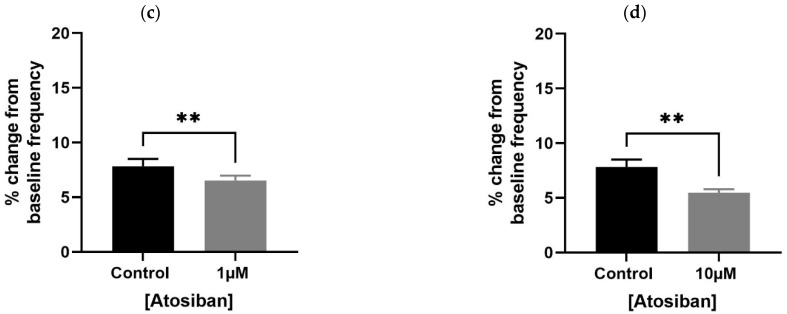
Atosiban reduces spontaneous bladder contractions frequency in obese and non-obese rats. Bar graphs show the percentage change in spontaneous bladder contraction frequency following treatment with atosiban (1 µM and 10 µM). Panels (**a**,**b**) represent data from obese rats (*n* = 6); panels (**c**,**d**) represent those from non-obese rats (*n* = 6). Values are expressed as mean ± SD relative to baseline. Significant reductions were observed in both groups (** *p* < 0.01; **** *p* < 0.0001; unpaired *t*-test).

**Figure 2:**
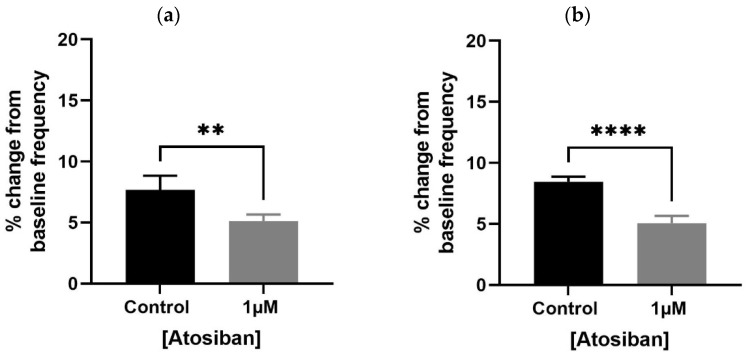
Atosiban reduces spontaneous prostate contractions frequency in obese and non-obese rats. Bar graphs show the percentage change in spontaneous contraction frequency in prostate tissues following atosiban (1 µM) treatment. (**a**) Obese rats; (**b**) non-obese rats (*n* = 6 per group). Values are expressed as mean ± SD relative to baseline. Significant reductions were observed in both groups (** *p* < 0.01; **** *p* < 0.0001; unpaired *t*-test).

Atosiban (1 µM) significantly reduced the frequency of spontaneous contractions in both the bladder and prostate. In obese rats, the reduction was pronounced in the bladder (**** *p* < 0.0001; Figure 1a,b) and significant in the prostate (** *p* < 0.01; Figure 2a). Similar effects were observed in non-obese rats, with significant reductions in both bladder (** *p* < 0.01; Figure 1c,d) and prostate (**** *p* < 0.0001; Figure 2b) tissues. Changes in the integral and maximum contraction amplitude following atosiban treatment were observed but did not reach statistical significance and are summarized in Supplementary Figures S1 and S2 for reference.

### 3.2. Oxytocin Enhances Bladder Contractility in Obese Rats

**Figure 3:**
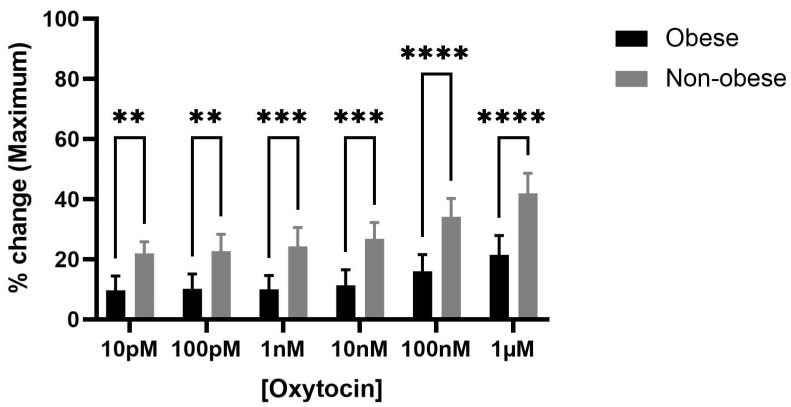
Oxytocin enhances maximum bladder contractile responses in obese versus non-obese rats. The bar graph shows the percentage change in maximum bladder contraction following cumulative oxytocin (OT) doses in obese and non-obese rats (*n* = 6 per group). Responses were normalized to 20 mM KCl-induced contraction. OT significantly increased the maximum contractile response in obese bladders compared to non-obese at all doses (**** *p* < 0.0001; *** *p* < 0.001; ** *p* < 0.01; 2-way ANOVA with Sidak’s post hoc test). Data are presented as mean ± SD.

Exposure to increasing concentrations of oxytocin (10 pM to 1 µM) induced a concentration-dependent increase in spontaneous bladder contractions in both obese and non-obese rats. Notably, the maximum baseline amplitude significantly increased in the bladder strips of obese rats compared to non-obese controls (**** *p* < 0.0001) (Figure 3). Although AUC (Supplementary Figure S3) and frequency (Supplementary Figure S4) showed an upward trend in both groups, these changes did not reach statistical significance.

### 3.3. Atosiban Attenuates Oxytocin-Induced Bladder Contractions

**Figure 4:**
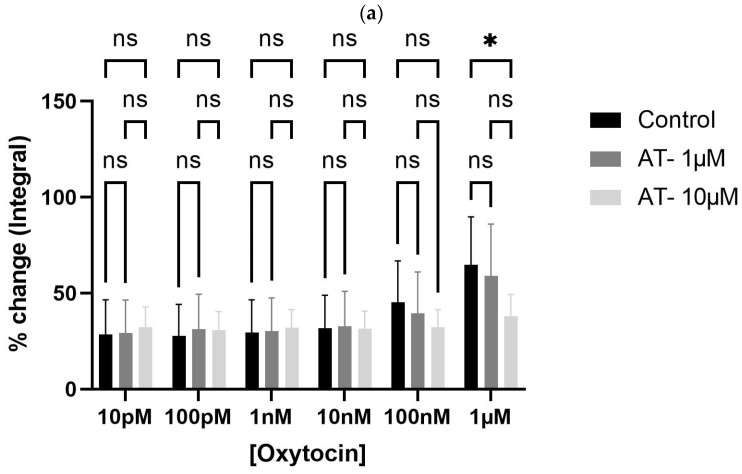

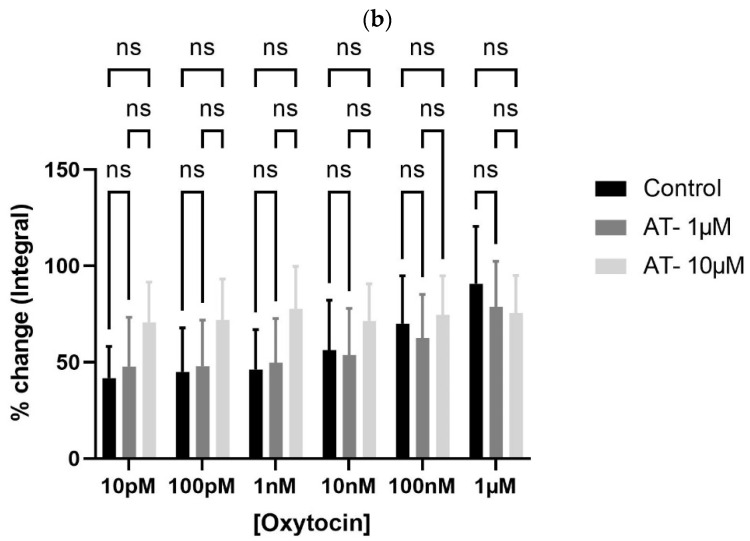
Atosiban reduces oxytocin-induced bladder contractility in obese rats. The bar graphs show the percentage change in area under the curve (AUC) of oxytocin-induced bladder contractions after atosiban (10 µM) treatment in obese (**a**) and non-obese (**b**) rats (*n* = 6 per group). Responses were normalized to 20 mM KCl-induced contractions. A significant reduction was observed in obese rats (* *p* < 0.05), with no significant effect in non-obese controls. Data are presented as mean ± SD (2-way ANOVA with Tukey’s post hoc test).

To test whether oxytocin’s effects were receptor-mediated, bladder tissues were pre-incubated with atosiban (1 µM and 10 µM). Atosiban (1 µM) significantly inhibited OT-induced bladder contractions (10 pM to 1 µM) in obese rats (* *p* < 0.05), particularly in terms of AUC (Figure 4a). These inhibitory effects were less pronounced in non-obese rats (Figure 4b).

### 3.4. Immunohistochemistry Findings

Immunohistochemical analysis was performed to assess oxytocin receptor (OXTR) expression and its cellular localization in bladder and prostate tissues from obese and non-obese rats (*n* = 6 per group). Dual immunofluorescence staining enabled the visualization and quantification of OXTR colocalization with smooth muscle actin, allowing for comparison between tissue compartments and experimental groups.

#### 3.4.1. OXTR Expression in Bladder

**Figure 5:**
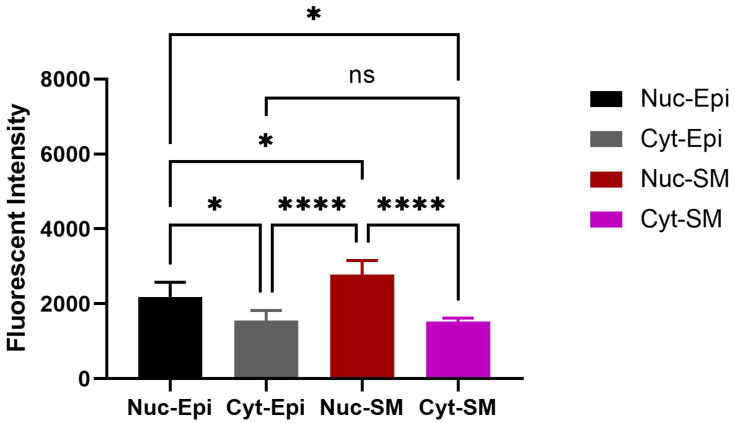
Quantitative analysis of nuclear OXTR in the bladder of obese animals. Nuclei show consistent OXTR staining in both epithelial and stromal (smooth muscle) regions. The graph depicts differences in nuclear OXTR intensity between epithelial and smooth muscle cells (*n* = 6; one-way ANOVA with Tukey’s multiple comparisons test). Statistical significance is indicated as follows: * *p* < 0.05, **** *p* < 0.0001. [Abbreviations: Nuc-Epi = Nuclear Epithelium; Nuc-SM = Nuclear Smooth Muscle; Cyt-Epi = Cytoplasmic Epithelium; Cyt-SM = Cytoplasmic Smooth Muscle].

**Figure 6:**
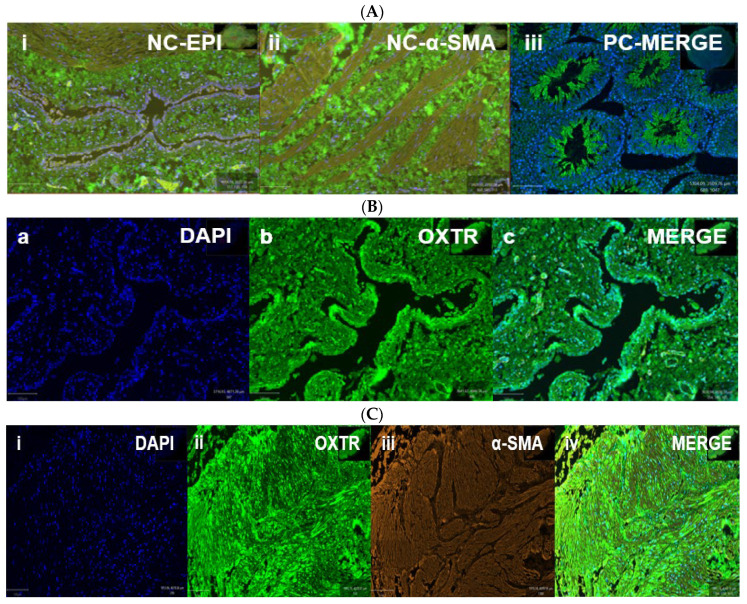
Oxytocin receptor (OXTR) expression in the bladder of obese rats. Immunofluorescence images show OXTR localization in bladder tissue from obese rats. Panel (**A**): Controls—(**A**-**i**,**A**-**ii**) negative controls without the primary antibody (epithelium and smooth muscle); (**A-iii**) positive control from testis tissue confirming OXTR specificity. Panel (**B**): Epithelial staining—(**B**-**a**) DAPI, (**B**-**b**) OXTR, (**B**-**c**) merged image showing OXTR in epithelial cells. Panel (**C**): Smooth muscle staining—(**C**-**i**) DAPI, (**C**-**ii**) OXTR, (**C**-**iii**) α-SMA, and (**C**-**iv**) merged image indicating OXTR colocalization with smooth muscle. Scale bar = 100 µm. [Abbreviations: NC = negative control; PC = positive control; OXTR = oxytocin receptor; α-SMA = alpha smooth muscle actin; DAPI = nuclear stain].

OXTR expression was identified in both epithelial and stromal compartments of the bladder, with predominant nuclear localization observed in both epithelial and smooth muscle cells (Figure 5, * *p* < 0.05). Double immunofluorescence revealed the colocalization of OXTR with α-smooth muscle actin (α-SMA) in smooth muscle layers, confirming receptor presence on contractile tissue (Figure 6).

#### 3.4.2. OXTR Expression in Prostate

**Figure 7:**
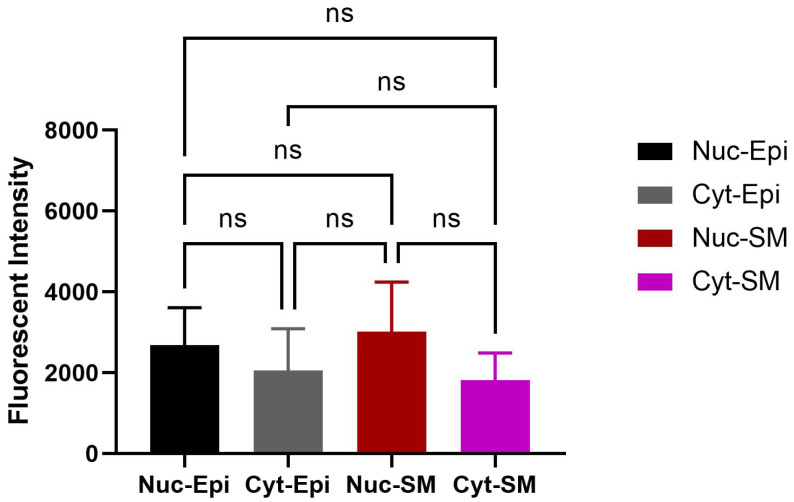
Quantitative analysis of nuclear OXTR in the prostate of obese rats. Nuclear OXTR intensity was compared between epithelial and smooth muscle regions. Although slightly higher in epithelial cells, the difference was not statistically significant (*n* = 6; unpaired *t*-test, *p* ≥ 0.05). Data are shown as mean ± SD. Abbreviations: Nuc-Epi = nuclear epithelium; Nuc-SM = nuclear smooth muscle; Cyt-Epi = cytoplasmic epithelium; Cyt-SM = cytoplasmic smooth muscle.

**Figure 8:**
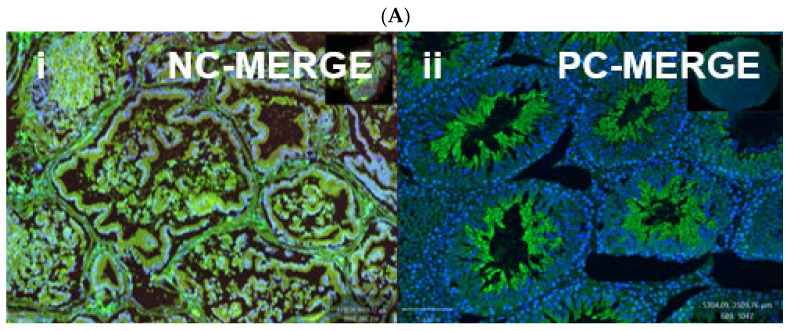

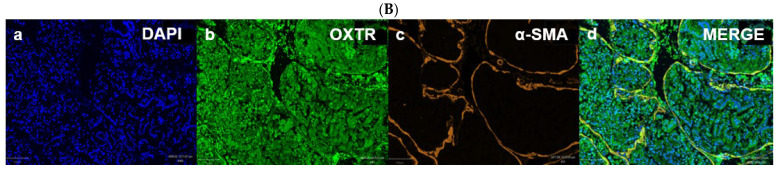
Immunofluorescent localization of oxytocin receptor (OXTR) in the prostate tissue of obese rats. Panels (**A**-**i**) and (**A**-**ii**) show negative and positive controls, confirming the specificity of OXTR immunostaining in prostate sections. Panels (**B**-**a**) to (**B**-**d**) illustrate immunofluorescence images of the prostate: (**B**-**a**) DAPI nuclear stain, (**B**-**b**) OXTR staining, (**B**-**c**) α-smooth muscle actin (α-SMA) indicating smooth muscle, and (**B**-**d**) merged image showing OXTR distribution predominantly within the glandular epithelium. All images were acquired under identical exposure settings. Scale bar = 100 µm. [Abbreviations: NC-MERGE = negative control merge; PC-MERGE = positive control merge; DAPI = 4′,6-diamidino-2-phenylindole; OXTR = oxytocin receptor; α-SMA = alpha smooth muscle actin; MERGE = merged image].

Like the bladder, nuclear-specific OXTR expression was observed within the epithelial and stromal regions of the prostate (Figure 7). Double immunofluorescence staining revealed colocalization of OXTR with α-SMA in smooth muscle cells (Figure 8), although this did not reach statistical significance (*p* > 0.05). These findings support a possible modulatory role of OT in prostate contractility.

## 4. Discussion

This study is the first to investigate and compare the effects of oxytocin (OT) and its antagonist atosiban (AT) on both spontaneous and OT-induced contractions in the bladder and prostate of obese and non-obese male rats. Our findings demonstrate that OT significantly increased bladder contractions in obese rats, and these effects were markedly attenuated by AT. In contrast, the effects of OT and AT on the prostate were minimal and did not reach statistical significance, suggesting a tissue-specific response and potentially altered receptor sensitivity between organs. This study showed that OT significantly increased the baseline amplitude of spontaneous bladder contractions in obese rats but not in non-obese rats. Although no significant changes were seen in contraction frequency, a trend towards increased area under the curve (AUC) was noted in both groups. These results align with those of Romine et al. (1985), who reported OT-induced detrusor contractions in male rabbits [39]. However, these findings differ from those noticed by Trahan et al. (2020), who observed no such effects in human detrusor muscle, highlighting possible interspecies differences [40].

The current study also observed that spontaneous contractions are not typically dominant in isolated bladder and prostate tissues under basal conditions; they were consistently recorded in our organ bath setup across all groups. Similar observations have been reported by Assinder and Nicholson (2007) [41]. Furthermore, the observed reduction in spontaneous contraction frequency following AT treatment further supports the notion that endogenous OT contributes to smooth muscle tone in these tissues. This is consistent with previous studies demonstrating paracrine OT activity and the presence of pacemaker-like interstitial c-Kit+ cells in the prostate, which generate slow-wave activity similar in profile to spontaneous contractions. These findings suggest a potential myogenic basis for the contractile patterns observed [42,43].

The present study also noticed some modulatory effects of OT on spontaneous contractions and of atosiban on OT-induced contractions in the prostate of both obese and non-obese rats. However, OT and atosiban did not significantly alter the frequency of OT-induced contractions in the prostate. This contrasts with findings by Lee et al. (2021), who reported that OT significantly increased the frequency of spontaneous contractions in the human prostate, with atosiban attenuating this response [42]. These discrepancies likely reflect interspecies differences in prostate physiology and tissue architecture [44].

Our immunofluorescence analysis revealed the robust nuclear-specific expression of oxytocin receptors (OXTR) in both bladder and prostate tissues, with higher intensity in the smooth muscle compartment, particularly in obese rats. This supports a role for OXTR in mediating the observed OT-induced contractile effects and is consistent with previous reports showing nuclear localization and age-related increases in OXTR expression [42,45,46].

The present study provides novel evidence that oxytocin enhances bladder contractility in obese rats and that atosiban can attenuate this effect. Immunohistochemical analysis further revealed increased OXTR expression in bladder tissues from obese rats, adding mechanistic insight into obesity-related bladder dysfunction. While these findings advance the current understanding of the potential role of OT–OXTR signaling in the lower urinary tract, the translational impact should be interpreted with caution. The relatively small sample size, absence of functional nerve-mediated experiments, and lack of hormonal profiling—particularly testosterone measurement—limit the ability to fully extrapolate these results to human pathophysiology. Future studies incorporating larger cohorts, neurotransmitter-driven contractility assays, and endocrine assessments are warranted to strengthen translational relevance.

## 5. Conclusions

This study provides novel evidence that oxytocin enhances bladder contractility, particularly in obese rats, potentially via nuclear-localized oxytocin receptors. Atosiban effectively attenuated these effects in the bladder but had minimal impact on the prostate.

Although spontaneous contractions were used as of smooth muscle tone, their reduction following atosiban treatment supports a role for endogenous oxytocin in maintaining baseline activity. These findings suggest that oxytocin signaling may contribute to alterations in lower urinary tract function. Further studies incorporating hormonal profiling and functional assays involving neurotransmitter-mediated responses are warranted to fully elucidate the mechanisms and potential translational relevance of oxytocin signaling in obesity-related bladder dysfunction.

## Data Availability

Data is unavailable due to privacy or ethical restrictions.

## Supplementary Materials

The following supporting information can be downloaded at: https://www.mdpi.com/article/10.3390/biomedicines13092097/s1, Figure S1: Effects of atosiban on spontaneous bladder contractions in obese and non-obese rats. Bar graphs illustrate the effects of atosiban at 1 µM and 10 µM on the percentage change in baseline integral and maximum contraction values. Panels (a) and (b) represent obese rats, and panels (c) and (d) correspond to non-obese rats (*n* = 5 per group). Atosiban (1 µM) reduced both parameters in both groups, but these changes were not statistically significant (*p* ≥ 0.05). However, atosiban at 10 µM significantly reduced the integral of spontaneous contractions in obese bladders (panel a). Data are shown as mean ± SD, with statistical comparisons made using unpaired two-tailed *t*-tests; Figure S2: Effects of Atosiban (1 µM) on Spontaneous Prostate Contractions in Obese and Non-Obese Rats. Bar graphs depict the percentage change from baseline in integral and maximum values of spontaneous prostate contractions following treatment with atosiban (1 µM). Panels (a) and (b) show data from obese rats, indicating a decrease in both parameters, whereas panels (c) and (d) present data from non-obese rats, with no significant changes observed. All reductions were not statistically significant (*p* ≥ 0.05, unpaired two-tailed *t*-test; *n* = 5 per group). Data are expressed as mean ± SD; Figure S3: Effects of Oxytocin on Area Under the Curve (AUC) of Bladder Contractions in Obese and Non-Obese Rats. Bar graph showing the percentage change in AUC of spontaneous bladder contractions in response to cumulative doses of oxytocin (OT) in obese and non-obese rats (*n* = 5 per group). Both groups exhibited a trend towards increased AUC, with a greater but non-significant response in non-obese rats compared to obese rats. Contraction responses were normalized to the response induced by 20 mM potassium chloride (KCl). Data are presented as mean ± SD. Statistical analysis was conducted using 2-way ANOVA with Sidak’s multiple comparisons test (*p* > 0.05). Error bars represent standard deviation; Figure S4: Effects of Exogenous Oxytocin on Frequency of Spontaneous Bladder Contractions in Obese and Non-Obese Rats. Graph depicting the percentage change in frequency of spontaneous bladder contractions following cumulative doses of oxytocin in obese and non-obese rats (*n* = 5 per group). No significant differences were observed between the groups. Responses were normalized to contractions induced by 20 mM potassium chloride (KCl). Data are presented as mean ± SD. Statistical analysis was performed using 2-way ANOVA with Sidak’s multiple comparisons test (*p* > 0.05).

## List of Abbreviations

**Abbreviation**	**Meaning**
LUTS	Lower Urinary Tract Symptoms
OAB	Overactive Bladder
BPH	Benign Prostatic Hyperplasia
OT	Oxytocin
AT	Atosiban
OXTR	Oxytocin Receptor
MetS	Metabolic Syndrome
IHC	Immunohistochemistry
SMC	Smooth Muscle Cell
α-SMA	Alpha-Smooth Muscle Actin
AUC	Area Under the Curve
IP_3_	Inositol Triphosphate
DAG	Diacylglycerol
PLC	Phospholipase C
DAPI	4′,6-Diamidino-2-Phenylindole
LH	Luteinizing Hormone
FSH	Follicle-Stimulating Hormone
HDL-C	High-Density Lipoprotein Cholesterol
PDE-5 Inhibitors	Phosphodiesterase Type 5 Inhibitors
