# Supplementary material for: Investigating the Pharmacological Impact of Atosiban, an Oxytocin Receptor Antagonist, on Bladder and Prostate Contractions Within OBESE and Non-Obese Rats
Source: Biomedicines. 2025 Aug 28;13(9):2097. doi: 10.3390/biomedicines13092097 (PMC12467195; doi:10.3390/biomedicines13092097)
Supplement: Supplementary file 1 [file biomedicines-13-02097-s001.zip › biomedicines-3742046-supplementary.pdf]

biomedicines

Supplementary Figures

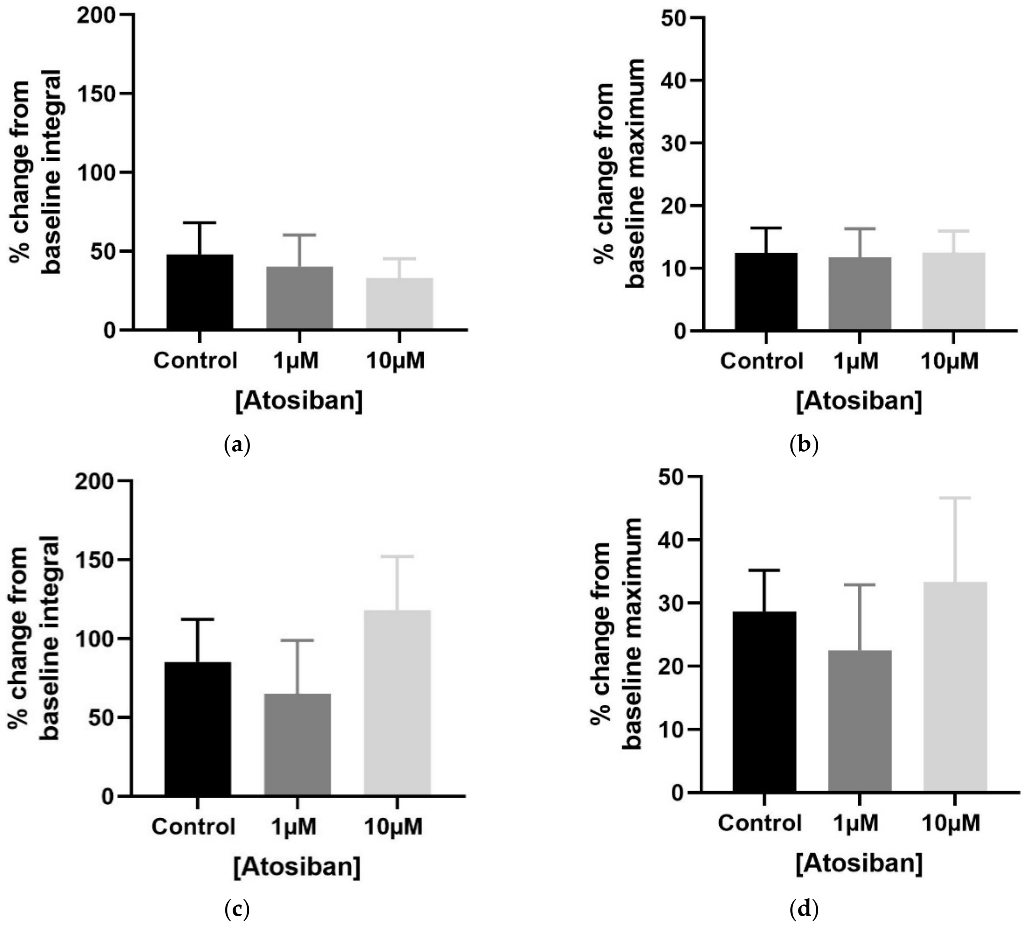

Figure S1. Effects of atosiban on spontaneous bladder contractions in obese and non-obese rats. Bar graphs illustrate the effects of atosiban at 1 µM and 10 µM on the percentage change in baseline integral and maximum contraction values. Panels (a) and (b) represent obese rats, and panels (c) and (d) correspond to non-obese rats (n = 5 per group). Atosiban (1 µM) reduced both parameters in both groups, but these changes were not statistically significant ($p \geq 0.05$). However, atosiban at 10 µM significantly reduced the integral of spontaneous contractions in obese bladders (panel a). Data are shown as mean ± SD, with statistical comparisons made using unpaired two-tailed *t*-tests.

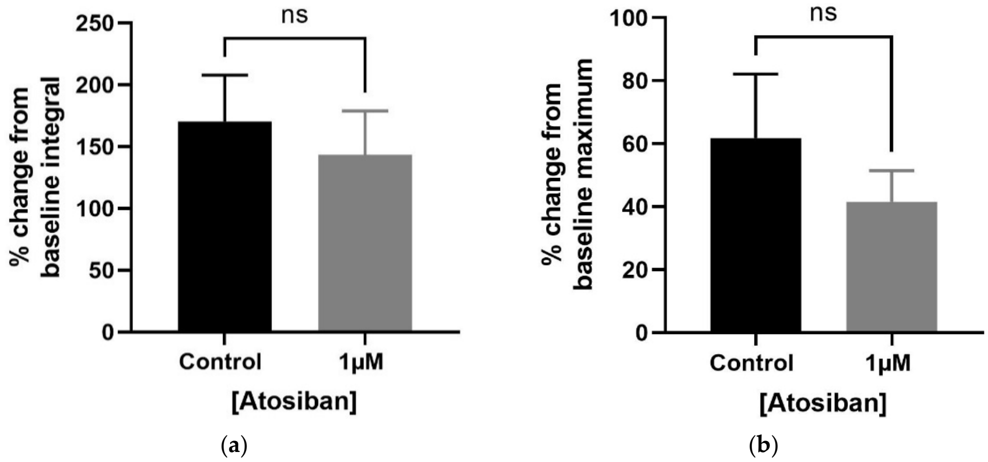

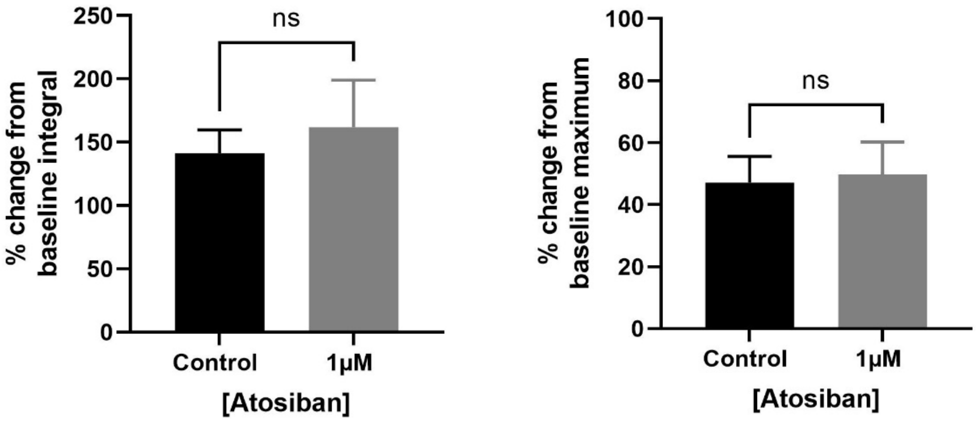

(c) (d)

Figure S2. Effects of Atosiban (1 μM) on Spontaneous Prostate Contractions in Obese and Non-Obese Rats. Bar graphs depict the percentage change from baseline in integral and maximum values of spontaneous prostate contractions following treatment with atosiban (1 μM). Panels (a) and (b) show data from obese rats, indicating a decrease in both parameters, whereas panels (c) and (d) present data from non-obese rats, with no significant changes observed. All reductions were not statistically significant ($p \geq 0.05$, unpaired two-tailed t-test; n = 5 per group). Data are expressed as mean ± SD.

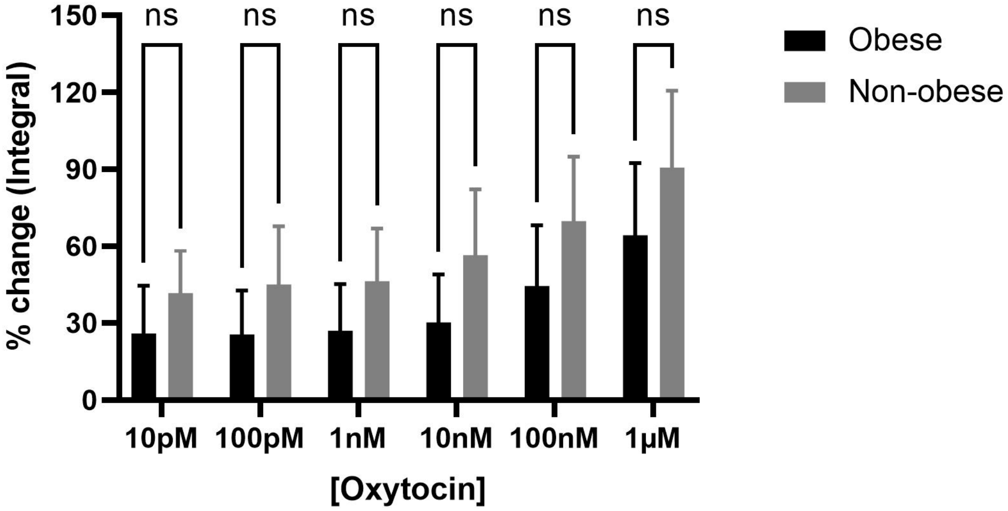

Figure S3. Effects of Oxytocin on Area Under the Curve (AUC) of Bladder Contractions in Obese and Non-Obese Rats. Bar graph showing the percentage change in AUC of spontaneous bladder contractions in response to cumulative doses of oxytocin (OT) in obese and non-obese rats (n = 5 per group). Both groups exhibited a trend towards increased AUC, with a greater but non-significant response in non-obese rats compared to obese rats. Contraction responses were normalized to the response induced by 20 mM potassium chloride (KCl). Data are presented as mean ± SD. Statistical analysis was conducted using 2-way ANOVA with Sidak's multiple comparisons test ($p > 0.05$). Error bars represent standard deviation.

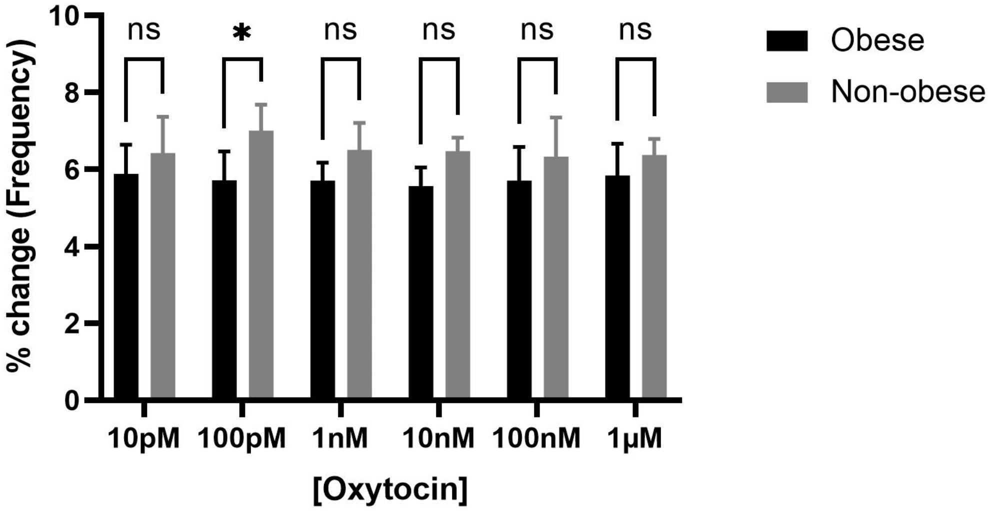

Figure S4. Effects of Exogenous Oxytocin on Frequency of Spontaneous Bladder Contractions in Obese and Non-Obese Rats. Graph depicting the percentage change in frequency of spontaneous bladder contractions following cumulative doses of oxytocin in obese and non-obese rats (n = 5 per group). No significant differences were observed between the groups. Responses were normalized to contractions induced by 20 mM potassium chloride (KCl). Data are presented as mean ± SD. Statistical analysis was performed using 2-way ANOVA with Sidak's multiple comparisons test ($p > 0.05$).
